# cnnLSV: detecting structural variants by encoding long-read alignment information and convolutional neural network

**DOI:** 10.1186/s12859-023-05243-x

**Published:** 2023-03-28

**Authors:** Huidong Ma, Cheng Zhong, Danyang Chen, Haofa He, Feng Yang

**Affiliations:** 1 School of Computer, Electronics and Information, Guangxi University, Nanning, 530004 China; 2 Key Laboratory of Parallel, Distributed and Intelligent Computing of Guangxi Universities and Colleges, Guangxi University, Nanning, 530004 China

**Keywords:** Structural variant detection, Long reads, Encoding alignment information, Convolutional neural network

## Abstract

**Background:**

Genomic structural variant detection is a significant and challenging issue in genome analysis. The existing long-read based structural variant detection methods still have space for improvement in detecting multi-type structural variants.

**Results:**

In this paper, we propose a method called cnnLSV to obtain detection results with higher quality by eliminating false positives in the detection results merged from the callsets of existing methods. We design an encoding strategy for four types of structural variants to represent long-read alignment information around structural variants into images, input the images into a constructed convolutional neural network to train a filter model, and load the trained model to remove the false positives to improve the detection performance. We also eliminate mislabeled training samples in the training model phase by using principal component analysis algorithm and unsupervised clustering algorithm *k*-means. Experimental results on both simulated and real datasets show that our proposed method outperforms existing methods overall in detecting insertions, deletions, inversions, and duplications. The program of cnnLSV is available at https://github.com/mhuidong/cnnLSV.

**Conclusions:**

The proposed cnnLSV can detect structural variants by using long-read alignment information and convolutional neural network to achieve overall higher performance, and effectively eliminate incorrectly labeled samples by using the principal component analysis and *k*-means algorithms in training model stage.

**Supplementary Information:**

The online version contains supplementary material available at 10.1186/s12859-023-05243-x.

## Background

According to the number of base pairs (bp) changed, genomic variants can be divided into single-nucleotide polymorphisms (SNPs), short insertions and deletions (Indels), and structural variants (SVs). Different from SNPs and Indels, SVs consist of more varied nucleotides. In addition, SVs often have a greater impact on organisms [1]. Various diseases have been confirmed to be closely related to genomic SVs [1, 2]. The accurate detection of multi-type SVs can provide data support for subsequent genomic analysis to uncover the relationship among SVs, gene expression and human evolution. However, the detection of structural variants still faces some challenges, such as inevitable errors from sequencing technologies and sequence alignment tools, and more complex SV types including insertions (INSs), deletions (DELs), inversions (INVs), duplications (DUPs), and translocations (TRAs) [3]. Therefore, genomic structural variant detection is an essential and challenging issue.

Compared with the short reads generated by next-generation sequencing (NGS) platforms, the long reads generated by the third-generation sequencing (TGS) technologies can not only more accurately align with the genome, but also cover entire SV and describe the breakpoints [4]. Existing studies have shown that long-read based callers perform better than short-read based callers in human genomic SV detection [4, 5]. Sniffles [6] first estimates and adjusts the parameters to fit the datasets used, then extracts the features of each SV and performs clustering to distinguish SVs types. The tool PBSV developed by PacBio [7] realigns abnormal reads to the reference genome to further analyze types of SVs. SVIM [8] transforms features from intra-alignment and inter-alignment information into graphs, and executes graph clustering method to classify SVs. CuteSV [9] extracts features of different kinds of SVs, uses feature similarity based unsupervised clustering method to cluster features and recognize the types of SVs, and applies clustering-and-refinement approach to improve the accuracy of breakpoints to reduce the number of false positives in detection results. In addition to the four classical multi-type SV detection methods mentioned above, there are also some callers for specific SV type or sequencing data, such as rMETL [10], NanoVar [11] and SKSV [12]. However, the performance of some existing methods using long-read alignment information depends on selection of features and parameters for detecting multiple types of SVs.

Convolutional neural network (CNN) [13] is a classical deep learning model commonly used for the analysis of two-dimensional data (e.g., images), and has been widely applied to researches in biomedical field such as skin segmentation [14], brain tumor classification [15], and diabetic retinopathy severity level prediction [16]. In recent years, deep learning models like CNN have also been applied to the detection of genomic variants similarly. By encoding alignment information for short reads to represent variant features, DeepVariant [17] applies the neural network to detect SNPs and Indels for the first time. DeepSV [18], DeepSVFilter [19] and Cnngeno [20] use the short-read alignment information to encode DELs and filter the detection results to improve accuracy. In Clairvoyante [21], a multi-task convolutional neural network model was proposed to overcome the impact of long-read sequencing errors on SNP detection and realize genotyping. InvBFM [22] and BreakNet [23] use support vector machine (SVM) and bidirectional recurrent neural network (BRNN) to detect INVs and DELs, respectively. And SVision [24], one deep learning based multi-object-recognition framework, was proposed to detect complex structural variants from long reads. If appropriate feature encoding strategy can be designed, the deep learning based detection method will autonomously learn the features of variants and have the fault-tolerant ability in detecting variants to a certain extent. Most of the existing deep learning based methods mainly focus on a specific type of variants. Hence, it is very promising to further investigate multi-type structural variant detection methods using long-read alignment information.

The main contributions of our work are as follows:

1. An encoding strategy for four types of structural variants is designed, and a method called cnnLSV is proposed to detect multi-type structural variants by encoding long-read alignment information and using convolutional neural network.
2. The cnnLSV integrates the initial results of existing callers to a callset to improve detection sensitivity, constructs a classification model to filter the obtained callset to eliminate false positives to improve detection precision.
3. To increase the training effect and prediction accuracy, the combined use of principal component analysis (PCA) [25] and unsupervised clustering algorithm *k*-means [26] is conducted to eliminate samples with incorrect labels in the training model stage.

The remainder of this paper is organized as follows. “Methods” section describes the proposed detection method in detail. “Results” section reports experimental results on the simulation and real datasets. “Discussion” section discusses the advantages and shortages of our proposed method. “Conclusion” section concludes the paper and gives future research directions.

## Methods

### Procedure of proposed method

**Fig. 1:**
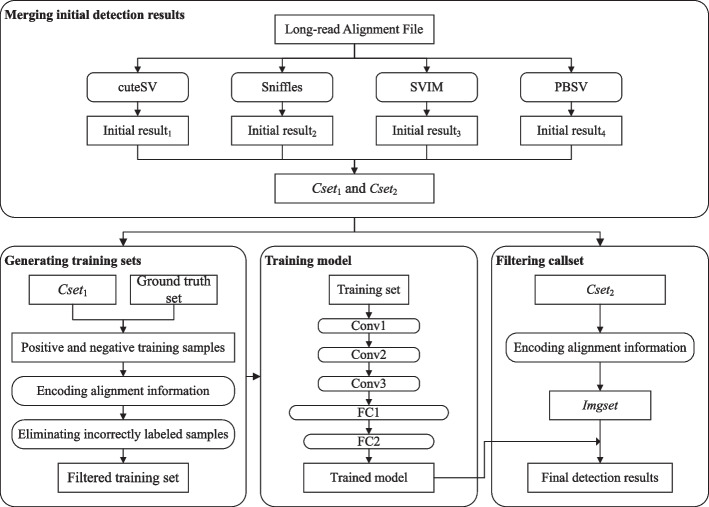
Excuting procedure of our proposed method. Conv and FC represent convolutional layer and fully connected layer, respectively

We propose a long-read based SVs detection method shown in Fig. 1.

The proposed method includes two stages of training filtering model and detecting structural variants. The initial datasets of the detection methods consist of the long-read alignment files, which are divided into dataset $Dset_{1}$ for training model and dataset $Dset_{2}$ for detecting. In the training model stage, we first execute existing callers on the $Dset_{1}$ to obtain initial detection results and merge the results into one callset $Cset_{1}$, compare $Cset_{1}$ with the ground truth set containing real variant information during the training model to obtain the training sets, and select true positives and false positives as the positive and negative training samples respectively. Secondly, we encode long-read alignment information around SVs into images according to the encoding methods related to different kinds of SVs. Thirdly, we execute the PCA and the *k*-means algorithms to eliminate the negative samples whose features are similar to that of positive samples due to label errors to obtain filtered training sets. Finally, the filtered training sets are input into CNN to train the classification model. In the SV detection stage, we first execute existing callers on the $Dset_{2}$ to obtain the initial detection results, and merge the results into another callset $Cset_{2}$. Secondly, we encode the long-read alignment information around SVs in $Cset_{2}$ into one image set *Imgset*. Finally, we use the trained model to filter *Imgset* to obtain the final detection results.

### Encoding long-read alignment information

The performance of some existing long-read based SVs detection methods is mainly affected by some parameters. For example, one important parameter is the minimal number of supporting reads in Sniffles, SVIM, and cuteSV. If the value of the minimal number of supporting reads is too large, the detection results will achieve high precision but low recall; and if its value is too small, the opposite is true. In addition, different types of SVs are also suitable for different parameter values. This means that users will face the problem of selecting parameters. Therefore, we encode the long-read alignment information around the variant region into images according to the variant features, and then assign a large number of images containing positive and negative examples to the CNN for training the filtering model. For a new image generated by a variant, the trained model will autonomously judge whether the variant is the true positive or false positive. The image describes the arrangement of reads with variant characteristics. The deep learning based detection method has fault tolerance ability to select the features of variants.

**Fig. 2:**
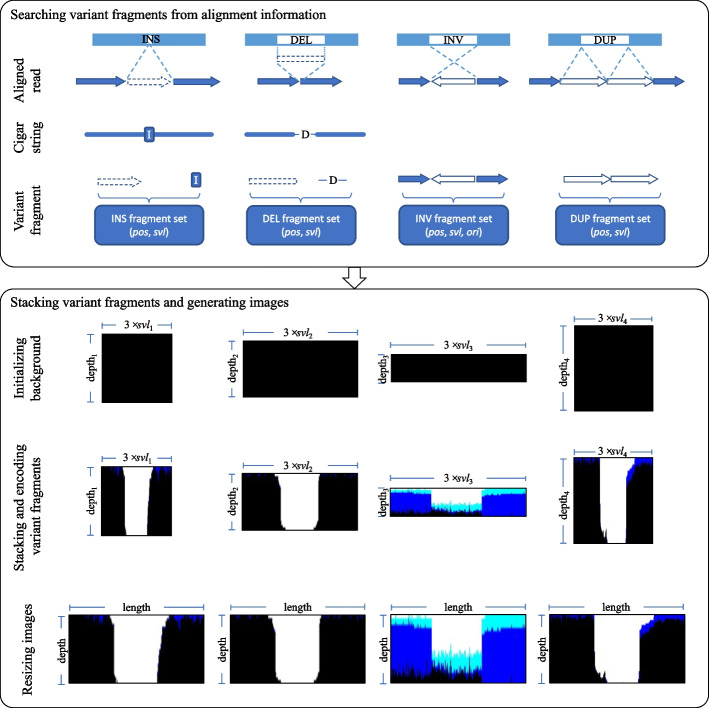
Process of encoding long-read alignment information , where *pos*, *svl*, *ori* represent the position, length, and the orientation of the variant fragment, respectively

According to the characteristics of SVs and long-read sequencing data, we present an improved encoding method. The encoding method determines the range of searching long-read alignment information according to the length of SV. In this way, we can obtain enough read alignment information for variants of any length to generate images. The images generated by real variants of different lengths will have similar features, which is conducive to improve the training effect of the subsequent binary classification model. Compared with the existing deep learning based encoding method, our improved encoding method only represents those read fragments with the feature of variant in images. It is well known that INSs and DELs are the two most abundant SVs in the genome. For the fragments where INSs or DELs occur frequently, the two adjacent variants are too close to each other to fully represent the true positive features. Our encoding method dynamically adjusts the image to a uniform size according to the coverage of each variant fragment. By visualizing the long-read alignment information in IGVtools, we found that there were different coverages in different fragments of the genome. Our method uses normal alignment information to calculate the coverage to dynamically adjust the depth of read stack to encode long-read alignment information in distinct fragments with different coverages. In order to obtain the images with the same size, our method scales each image according to search region, depth of read stack, and length and width of image. To distinguish the true positives and the false positives of the SVs in $Cset_{2}$, we propose feature extracting and image encoding methods for four variants INS, DEL, NVI, and DUP shown in Fig. 2. For the convenience of understanding our proposed encoding methods, we describe them in detail as follows.

#### Encoding for two variants INSs and DELs

We select $[bpt_{left}-svl, bpt_{right}+svl]$ and $[INS_{pos}-svl, INS_{pos}+2\times svl]$ as long-read alignment information search region for DELs and INSs respectively, where $bpt_{left}$ and $bpt_{right}$ denotes positions of the left and right breakpoints of DEL, $INS_{pos}$ is the position of INS, and *svl* is the length of SV. INS and DEL are characterized by the CIGAR string and split read alignment. For the features in the CIGAR string, we extract the variant fragments represented by “I” (INS) and “D” (DEL) in the CIGAR string of each read in search region, and obtain the starting and ending positions of each variant fragment. For split read alignment, if the distance between two segments from the same read increases or decreases over 50 bps before and after alignment, the genomes covered by these two segments may have DEL or INS variant. At this time, we obtain the starting and ending positions of the DEL or INS in the variant fragment. We assign values of three RGB channels to each variant fragment according to the following rules: if each variant fragment appears completely in the search region, the B channel is set to 255; if the midpoint for each variant fragment is between the two breakpoints, the G channel is set to 255; and if the length of each variant fragment is less than two times of *svl*, the R channel is set to 255.

#### Encoding for variant INVs

The lengths of some INVs will reach hundreds of thousands of bps. To accelerate encoding process for INV and reduce memory usage, we set a threshold $\alpha$ to determine the search region. If *svl* is less than or equal to $\alpha$, $[bpt_{left} - svl, bpt_{right} + svl]$ is used as the search region; if *svl* is greater than $\alpha$, SV information surrounding the left and right breakpoints is searched. The left and right search regions are $[bpt_{left} - \alpha , bpt_{left}+\alpha /2]$ and $[bpt_{right}+\alpha /2, bpt_{right}+\alpha ]$ respectively, where $bpt_{left}$ and $bpt_{right}$ denote positions of the left and right breakpoints of INV, and $\alpha$ represents the threshold length of INV. According to the characteristics of INV, the long reads in the search region are encoded by the following rules. If the read in this region is split into multiple segments, the B channel of each segment is set to 255; if the midpoint of each segment is between the two breakpoints, the G channel is set to 255; and if the orientation of each segment is inconsistent with that of other segments, the R channel is set to 255.

#### Encoding for variant DUPs

We select $[bpt_{left}-svl, bpt_{right}+svl]$ as long-read alignment information search region for DUPs, where $bpt_{left}$ and $bpt_{right}$ denote positions of the left and right breakpoints of DUP. Similar to the INV, DUP features are derived from split read alignment. If two segments of the same read overlap after the read is aligned to reference genome, the aligned segment region of reference genome may have tandem duplication. At this time, we achieve the starting and ending positions of the two segments. If each segment completely presents in the search region, the channel B is set to 255; if the midpoint of each segment is between the two breakpoints, the G channel is set to 255; if the variant length is less than two times of the *svl*, the R channel is set to 255.

### Training model and detecting SVs

#### Eleminating incorrectly labeled samples

**Fig. 3:**
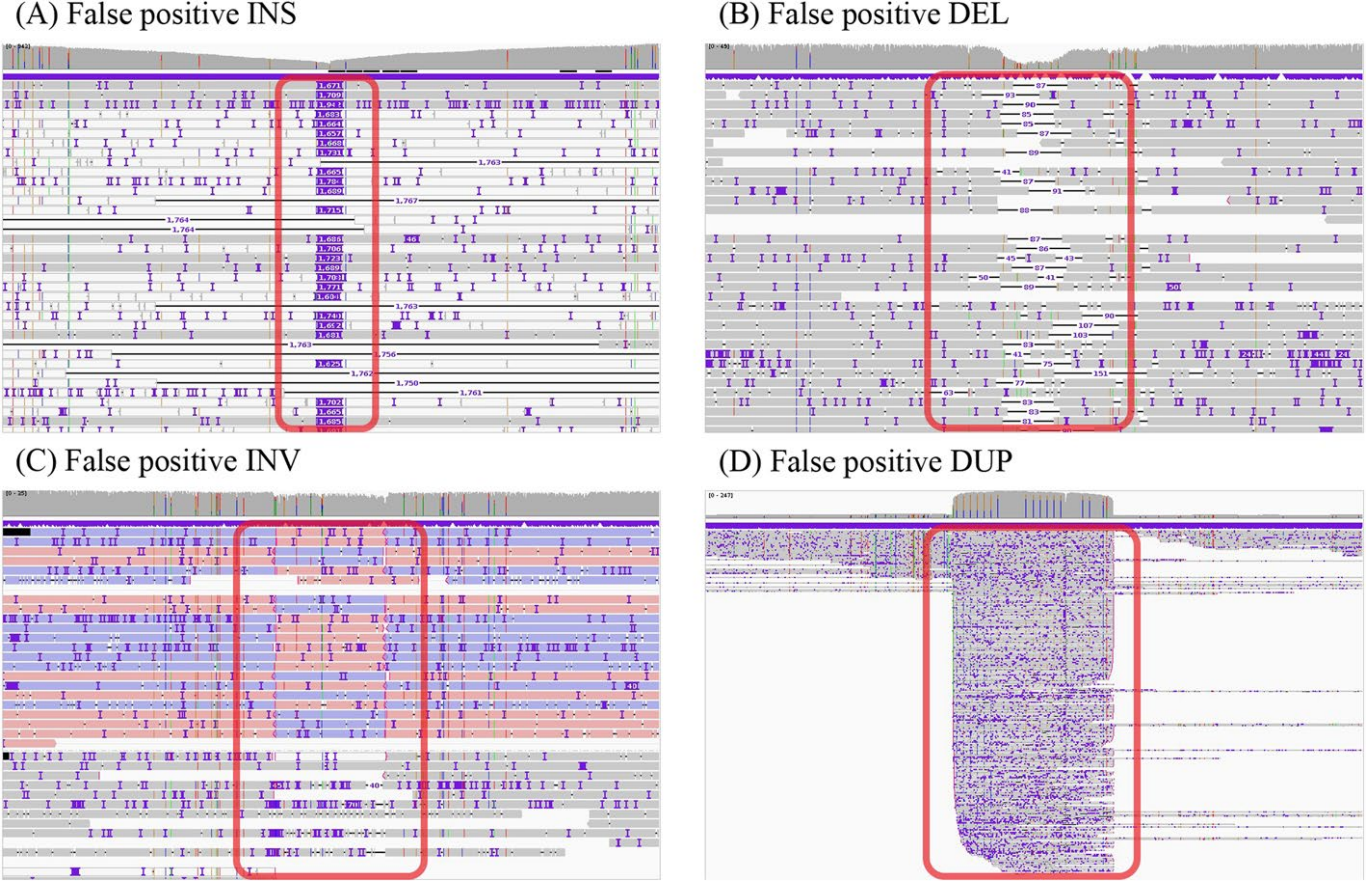
False positives with the same features as true positives in NA19240 dataset. **A** INS detected by Sniffles (5-17,587,397-1713), PBSV (5-17,587,396-1666), SVIM (5-17,587,396-1668), and cuteSV (5-17,587,397-1758). **B** DEL detected by Sniffles (7-4,179,134-85), PBSV (7-4,179,136-88), SVIM (7-4,179,144-87), and cuteSV (7-4,179,146-88). **C** INV detected by Sniffles (17-5,886,159-266), PBSV (17-5,886,159-266), SVIM (17-5,886,159-266), and cuteSV (17-5,886,163-272). **D** DUP detected by SVIM (9-140,739,516-1424) and cuteSV (9-140,739,517-1422). The content *chr*-*pos*-*svl* in parentheses represents the chromosome, position, and the length of the SV

Those structural variants detected by callers but not recorded in the ground truth sets are called false positives. However, the actual situation is that some false positives show the characteristics of true positives, which may be affected by the accuracy of aligners and the confidence of the ground truth sets. Figure 3 shows the long-read alignment information around some false positives. It illustrates that this part of false positives are labeled incorrectly. In order to prevent the model from judging true positives as false positives and filtering out them, we eliminate samples with incorrect labels by executing the PCA and *k*-means algorithms to improve the prediction accuracy of the filtering model.

**Fig. 4:**
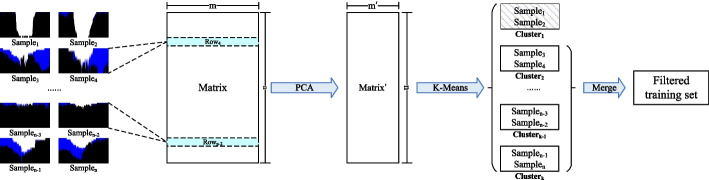
Eliminating incorrectly labeled samples in negative training samples

As shown in Fig. 4, in order to eliminate the incorrectly labeled samples in negative training samples, we first convert the 2-D RGB matrix parsed from the *i*-th image in set *Imgset* into an array $ArrE_{i}$ by the following formula:

1 $$\begin{aligned} \begin{aligned} ArrE_{ij}=\sum _{k=1}^{row}R_{kj}\times \omega ^{2}+G_{kj}\times \omega +B_{kj} \end{aligned} \end{aligned}$$

where $ArrE_{ij}$ represents the *j*-th element in $ArrE_{i}$, *row* is the number of rows of the RGB matrix, $R_{kj}$, $G_{kj}$, and $B_{kj}$ represent the values of three channels of the pixel point in the *k*-th row and *j*-th column in the RGB matrix respectively, *j*=1,2,..., *col*, and *col* is the number of columns of the RGB matrix, *i*=1,2,..., $|Imgset|$, $\omega$ is a control parameter that determines the converted value. The number of rows in *ArrE* represents the number of samples, and the number of columns in *ArrE* represents the number of features, *ArrE* =($ArrE_{1}, ArrE_{2},..., ArrE_{|Imgset|}$). Secondly, in order to improve the clustering effect, we use the PCA algorithm to reduce the redundant features to form a new matrix *ArrE*Â´ with less but important features. Thirdly, we execute the *k*-means algorithm for all row vectors in *ArrE*Â´ to obtain cluster $C_{l}$ whose vectors with similar features, $l=1,2,..,c$, where *c* denotes the number of clusters. Finally, we partition the *Imgset* into different sets *S* according to cluster $C_{l}$, and remove the sets with incorrectly labeled samples. Other remaining samples in *S* are used to form the final training set *S*Â´.

#### Training the filtering model

We construct a relatively lightweight neural network structure shown in Fig. 1. Each image in *S*Â´ is taken as the input of the constructed network. The CNN module of constructed network includes three identical structures, which each structure consists of a 3 $\times$ 3 $\times$ 3 convolution kernel, ReLU activation function, and a max-pooling layer. The matrix outputted by the CNN module is flattened into a vector and fed this vector into the classification module with two fully connected layers. Behind each fully connected layer, one dropout layer [27] is added to prevent overfitting. Since detecting SVs can be regarded as a binary classification problem, we select the most commonly used activation function sigmoid to calculate the output of the fully connected neural network. We use the images in training set *S*Â´ to train the model.

#### Detecting SVs on long-read datasets

The $Cset_{2}$ is used as the input of our proposed method cnnLSV. The long-read information for INS, DEL, INV, and DUP is encoded into the images. The trained model is used to determine whether the images represent true positives. If the final output value of the model is greater than 0.5, the image represents a true positive; otherwise, a false positive. Here, the true positive denotes the variant in long reads. We retain true positives and eliminate false positives of INS, DEL, INV and DUP in $Cset_{2}$. Different from the other four variants, the variant call format (VCF) file generated by callers records TRAs at the breakpoint level. It is difficult to encode TRAs occurring between two chromosomes into images. Thus, we do not filter the TRAs in $Cset_{2}$ by encoding alignment information and CNN, but combine the two TRAs $T_{a}$ and $T_{b}$ in $Cset_{2}$ representing the same TRA variant into one TRA. $T_{a}$ and $T_{b}$ are considered to be the same TRA by the formulas (2) and (3):

2 $$\begin{aligned}{} & {} Chr_{a1}=Chr_{b1}\ and\ Chr_{a2}=Chr_{a2} \end{aligned}$$

3 $$\begin{aligned}{} & {} |Pos_{a1}-Pos_{b1}|\le \gamma \ and\ |Pos_{a2}-Pos_{b2}|\le \gamma \end{aligned}$$

where $Chr_{i1}$ and $Chr_{i2}$ represent the two chromosomes where the fragment exchange occurred in the *i*-th TRA variant information, $Pos_{i1}$ and $Pos_{i2}$ denote the breakpoint positions of the two chromosomes, *i*=a, b, and $\gamma$ indicates the threshold of the bias of the $Pos_{aj}$ and $Pos_{bj}$ and its default value is 1000, *j*=1, 2. After combining the variant information representing the same TRA into the same cluster, we select the TRAs detected by at least $c_{0}$ detection methods as the final output, $c_{0}$ and $C_{sup}$ meet the following constraint:

4 $$\begin{aligned} \begin{aligned} C_{sup}\ge c_{0} \end{aligned} \end{aligned}$$

where $C_{sup}$ is the number of callers that detected the TRAs.

#### Algorithm

Let *GTset* denote the ground truth set corresponding to $Dset_{1}$, *VCFfile* represent the variant call format file with detection results, *Img* be the image set for training, *img* represent an image generated by variant, *Bset* be the set for storing TRA, *chr*, *pos*, *svl*, and *svt* represent the chromosome, position, length and the type of the *i*-th SV respectively, $Arrs_{1}$ denote the set of one-dimensional arrays, $Arrs_{2}$ represent the set of low dimensional arrays. By encoding long-read alignment information and using CNN, Algorithm 1 describes our proposed detecting four-type structural variant algorithm called cnnLSV.

**Figure:**
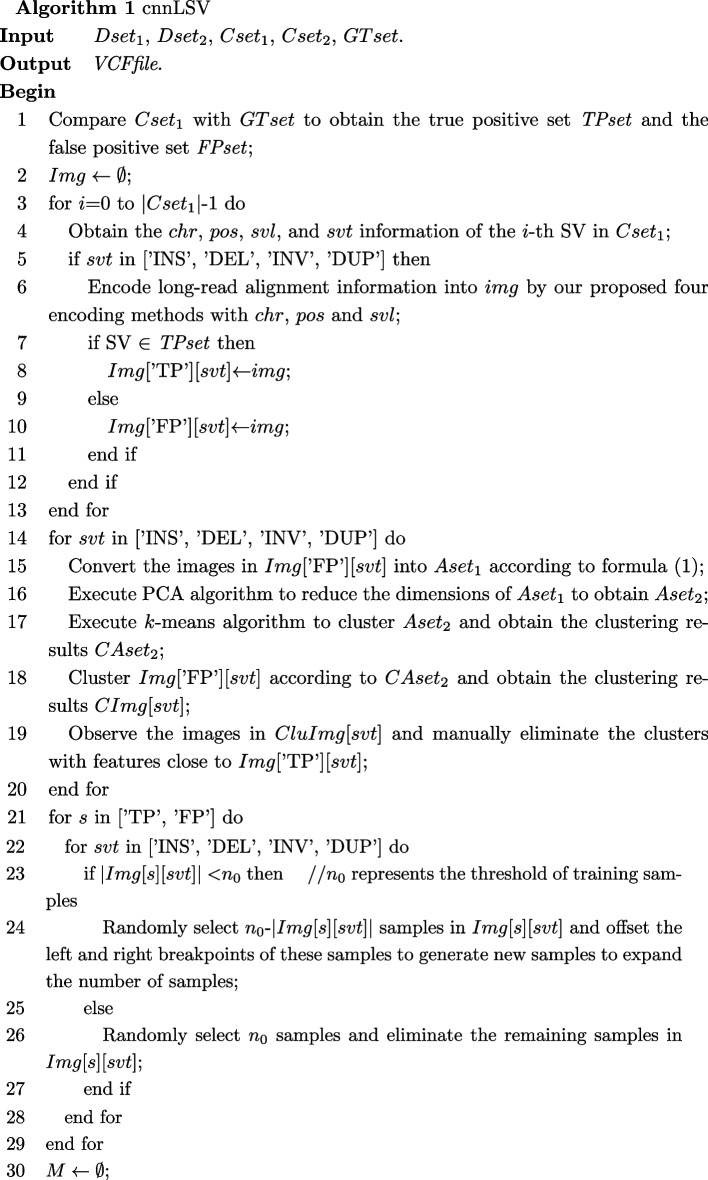


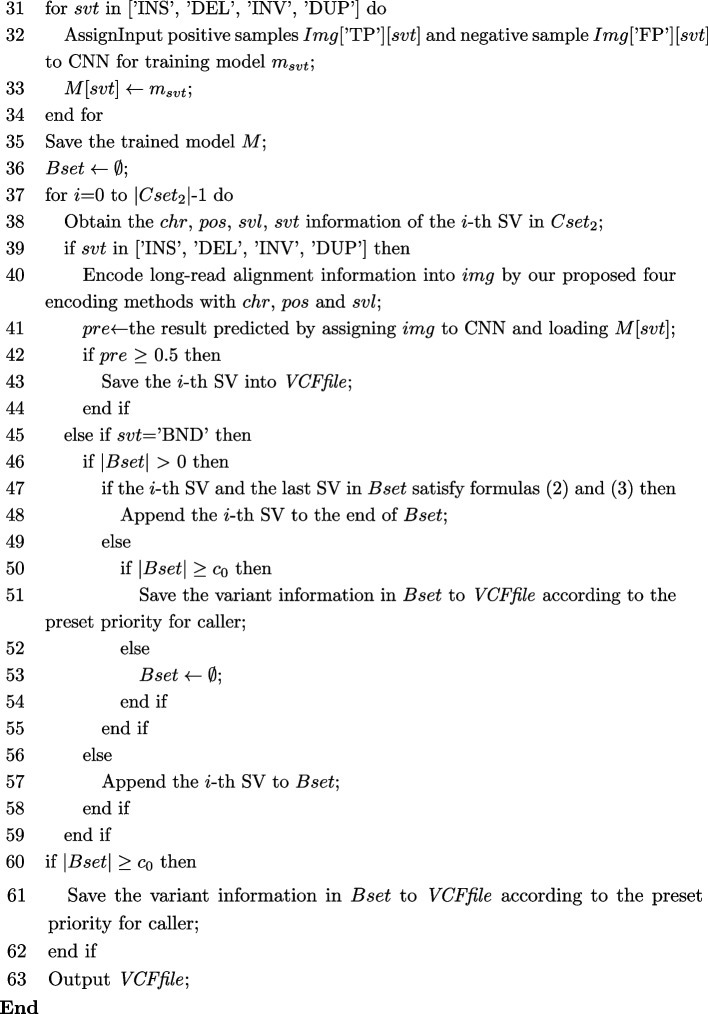


## Results

### Experimental environment

The experiment was carried out on the computing node X580-G30 with CPU 2 $\times$ Intel Xeon Gold 6230, GPU 2 $\times$ Tesla T4, and main memory 192GB DDR4 of Sugon 7000A parallel computer cluster system at Guangxi University. The running operating system is CentOS 7.4. The proposed method was implemented by Python3.8 programming. The Pytorch was used to train and test the constructed network model.

### Experiment on simulated datasets

**Fig. 5:**
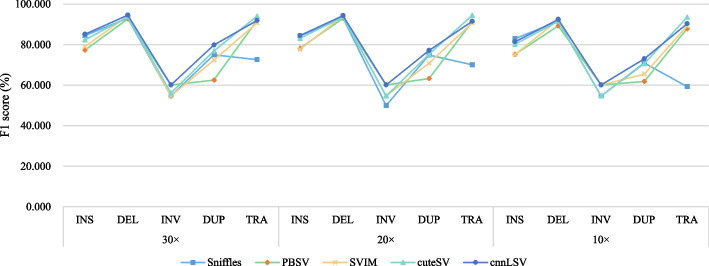
Detection results on simulated datasets

We obtained the real variants provided by cuteSV, used the tool Visor [28] to insert these SVs to some specific positions in the reference genome, and applied the tool PBSIM [29] to generate the long-read alignment files with 30 $\times$ coverage for each type of SV. We detected the simulated datasets by executing methods Sniffles, PBSV, SVIM, and cuteSV, and used the Bcftools [30] to merge the detected results of the four methods into a set *Meg*. We extracted the variant information of chromosomes with no.1-16 from *Meg* as a training set *Me*$g_{t}$ for training model, and extracted the variants of the remaining chromosomes from *Meg* as a set *Me*$g_{d}$ for evaluating the performance of the detection methods. Let $M_{30}$ denote the model trained on the datasets with 30 $\times$ coverage. By executing our method cnnLSV, we obtained $M_{30}$ trained with *Me*$g_{t}$ and filtered the false positives of INS, DEL, INV and DUP in *Me*$g_{d}$ to achieve the final detection results. To verify the impact of the coverage on detection performance and generality of the model $M_{30}$, we also generated 20 $\times$ and 10 $\times$ datasets $D_{20}$ and $D_{10}$, and used $M_{30}$ to filter the detection results of executing methods Sniffles, PBSV, SVIM, and cuteSV on the two datasets $D_{20}$ and $D_{10}$. We used the F1-score to balance precision and recall as the final detection performance metric. Precision *Pre*, recall *Rec*, and F1-score *F*1 are computed by the following formulas:

5 $$\begin{aligned}{} & {} Pre=\frac{TP}{TP+FP} \end{aligned}$$

6 $$\begin{aligned}{} & {} Rec=\frac{TP}{TP+FN} \end{aligned}$$

7 $$\begin{aligned}{} & {} F1=\frac{2\times Pre\times Rec}{Pre+Rec} \end{aligned}$$

where *TP*, *FP*, and *FN* represent the number of true positives, false positives, and false negatives, respectively. The detection results on the simulated datasets are shown in Fig. 5.

As shown in Fig. 5, when the five methods were executed on the 30 $\times$ and 20 $\times$ datasets, our method cnnLSV achieved the highest F1-scores for INS/DEL, INV, and DUP, and the second high F1-score for TRA which was a bit lower than that of cuteSV. When executing the five methods on the 10 $\times$ datasets, our method cnnLSV achieved higher F1-scores than other methods for detecting DEL, INV, and DUP, and the second high F1-scores for INS and TRA which were a little lower than Sniffles and cuteSV, respectively. The results indicate that cnnLSV achieves better detection performance overall. This is because cnnLSV filters all the detection results of the other four methods Sniffles, PBSV, SVIM, and cuteSV to reduce the false positives to achieve high precision and recall. In addition, it is noteworthy that our method cnnLSV used only one model $M_{30}$ to detect variants on 30 $\times$ , 20 $\times$ , and 10 $\times$ datasets and performed better on the whole. This illustrates that the detection model of cnnLSV has high adaptability because our proposed encoding method automatically calculates the depth of SVs and resizes the generated image to the uniform scale according to the depth in order to avoid the impact of the coverage as much as possible. The detailed detection results on simulated datasets for all five methods are provided in the supplementary materials (See: Table S1, S2, and S3 in Additional file 1).

### Experiment on real datasets

#### Datasets

In the experiment, we trained the filtering model on six PacBio datasets HG00512, HG00513, HG00731, HG00732, NA19238, and NA19239, and loaded the model to detect SVs on the five PacBio datasets HG00514, HG00733, NA19240 [31], HG002 CLR, and HG002 CCS [32]. Among them, the HG002 datasets are also used in Sniffles [6], PBSV [7] and cuteSV [9] to evaluate the performance of the detection methods. Table 1 shows the detailed information of all datasets with coverages and the average length of reads.

**Table 1:** Information of real datasets

*Datasets for training model*						
Dataset	HG00512	HG00513	HG00731	HG00732	NA19238	NA19239
Coverage	19 $\times$	18 $\times$	22 $\times$	23 $\times$	18 $\times$	16 $\times$
Average length	10,746	11,411	13,053	12,298	6801	6530
*Datasets for detecting SVs*						
Dataset	HG00514	HG00733	NA19240	HG002 CLR	HG002 CCS	
Coverage	41 $\times$	44 $\times$	37 $\times$	69 $\times$	28 $\times$	
Average length	11,800	12,295	6503	8323	13,478	

For the experiment on the datasets HG00514, HG00733, and NA19240, the minimal number of supporting reads and minimal SV length of Sniffles, SVIM, and cuteSV are set to 5 and 50 respectively, and these two parameters of PBSV follow its default settings. For the experiment on the datasets HG002 CLR 69 $\times$ , 40 $\times$ , 30 $\times$ , 20 $\times$ , 10 $\times$ , and HG002 CCS 28 $\times$ , 10 $\times$ , the minimal number of supporting reads of Sniffles and cuteSV is set to 10/5/4/4/3 and 3/2 respectively, and that of SVIM is set to 5/5/5/2/2 and 5/2 respectively. The minimal SV length of Sniffles, SVIM, and cuteSV is 50. The minimal number of supporting reads and the minimal SV length of PBSV also follow the default settings. We used the tool Truvari [33] to evaluate our proposed method cnnLSV with existing four long-read based SV detection methods Sniffles, PBSV, SVIM, and cuteSV by *Pre*, *Rec* and *F*1.

#### Training the model on real datasets

**Fig. 6:**
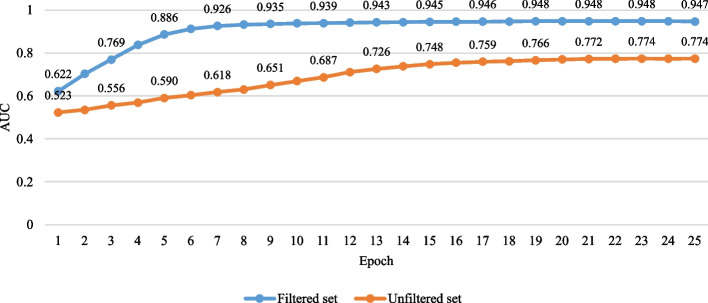
AUC curves during the training model stage. The blue and red curves represent training sets filtered and unfiltered by PCA and *k*-means algorithms, respectively

For each dataset, we first used Bcftools [30] to merge the initial detection results of executing Sniffles, PBSV, SVIM, and cuteSV into one callset and encoded INSs, DELs, INVs, and DUPS in the callset into images. We applied the PCA and *k*-means algorithms to eliminate the incorrectly labeled negative samples and selected 5000/5000/10000/10000 as the thresholds of INS/DEL/INV/DUP to balance the number of positive samples and the number of negative samples to improve effect of the training model. To verify the effect of executing algorithms PCA and *k*-means, we also balanced the positive samples and negative samples of images not filtered by PCA and *k*-means algorithms to train the constructed model. Figure 6 shows the AUC curves during the training model stage on unfiltered training sets and filtered training sets. It can be seen from Fig. 6 that the training result on the sets filtered by PCA and *k*-means algorithms has higher AUC values. This illustrates that the combing use of algorithms PCA and *k*-means can effectively reduce the number of incorrectly labeled samples to improve the effect of training model .

#### Detection results on real datasets

We first conducted the experiment on the first group of datasets including HG002 CLR and HG002 CCS. We used Samtools [30] to perform down-sampling on HG002 CLR and HG002 CCS datasets with different coverages to evaluate the performance of five detection methods. The experimental results are shown in Table 2.

**Table 2:** Performance of detection methods on datasets HG002 CLR and HG002 CCS

Coverage	Metric (%)	Sniffles	PBSV	SVIM	cuteSV	cnnLSV
*HG002 CLR*						
69 $\times$	*Pre*	94.364	94.825	94.981	95.233	**95.976**
	*Rec*	89.265	87.574	91.899	91.547	**93.403**
	*F*1	91.744	91.055	93.415	93.354	**94.672**
40 $\times$	*Pre*	93.127	94.782	95.343	95.253	**95.512**
	*Rec*	90.177	86.423	88.352	90.966	**91.754**
	*F*1	91.628	90.410	91.715	93.060	**93.595**
30 $\times$	*Pre*	91.710	95.001	**96.277**	94.941	95.143
	*Rec*	88.684	84.016	82.211	88.528	**89.410**
	*F*1	90.171	89.172	88.690	91.623	**92.187**
20 $\times$	*Pre*	92.845	95.427	85.530	**96.193**	92.835
	*Rec*	77.316	76.921	**90.374**	77.471	88.248
	*F*1	84.372	85.181	87.886	85.823	**90.483**
10 $\times$	*Pre*	90.322	**96.888**	91.837	96.365	92.161
	*Rec*	57.629	49.590	**73.685**	57.380	73.654
	*F*1	70.363	65.603	81.766	71.930	**81.875**
*HG002 CCS*						
28 $\times$	*Pre*	94.059	93.622	93.312	**94.964**	94.858
	*Rec*	**93.777**	84.172	92.770	93.393	93.673
	*F*1	93.918	88.646	93.040	94.172	**94.261**
10 $\times$	*Pre*	94.092	**95.468**	92.192	94.937	93.748
	*Rec*	88.373	75.532	90.343	87.014	**90.872**
	*F*1	91.143	84.338	91.258	90.803	**92.288**

The values in bold represent the best results

**Fig. 7:**
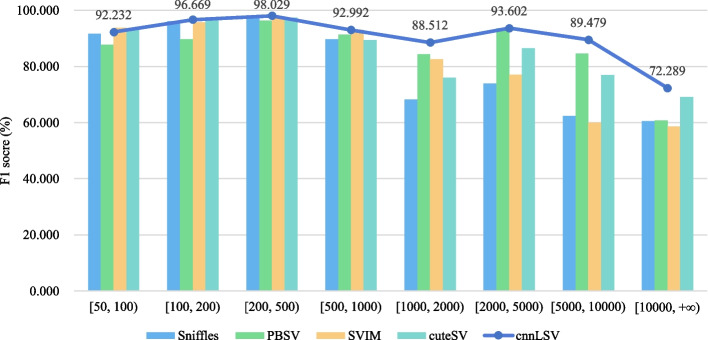
F1-scores of detection methods in detecting different lengths of SVs

We can see from Table 2 that our method cnnLSV performed best in all coverages on both HG002 CLR and HG002 CCS datasets among five methods. For the dataset HG002 CLR, the detection performance of each method was decreased as the coverage was decreased. For the HG002 CLR dataset with higher coverages 69 $\times$ and 40 $\times$ , cnnLSV achieved the highest precision, recall, and F1-score among five methods. Even for the HG002 CLR dataset with lower coverages, our method cnnLSV also achieved the highest F1-score. This is because cnnLSV can dynamically determine the depth of read stack and regions of searching long-read alignment information around each SV. It is noteworthy that all five methods performed better on the HG002 CCS 10 $\times$ dataset than on the HG002 CLR 10 $\times$ dataset. This indicates that the sequencing error rate has a significant impact on long-read SV detection. We also calculated F1-scores of all the methods in detecting SVs with different lengths on HG002 CLR. The statistical results are shown in Fig. 7. It can be seen intuitively from the Fig. 7 that cnnLSV got higher F1-scores than the other four methods in most cases, especially when the lengths of SVs exceed 1000. The detailed detection results on HG002 CLR and HG002 CCS datasets for all five methods are provided in the supplementary materials (See: Table S4 and S5 in Additional file 1).

In the following, we conducted the experiment on the second group datasets including HG00514, HG00733, and NA19240. We ran Sniffles, PBSV, SVIM, and cuteSV on the datasets in the second group, and merged their outputs as the input of our method cnnLSV. We compare the detection results of five methods with the ground truth set in Table 3.

**Table 3:** Performance of detection methods on datasets HG00514, HG00733, and NA19240

Type	Method	HG00514			HG00733			NA19240		
		*Pre* (%)	*Rec* (%)	*F*1 (%)	*Pre* (%)	*Rec* (%)	*F*1 (%)	*Pre* (%)	*Rec* (%)	*F*1 (%)
INS/DUP	Sniffles	43.278	60.934	50.610	34.907	61.731	44.596	42.219	60.949	49.883
	PBSV	50.588	53.652	52.075	50.254	53.960	52.041	51.152	52.469	51.802
	SVIM	44.559	61.158	51.555	42.574	62.097	50.515	45.762	61.180	52.359
	cuteSV	50.980	**66.202**	57.602	51.610	**68.674**	58.932	51.963	**63.710**	57.240
	cnnLSV	**57.539**	59.758	**58.628**	**59.114**	59.100	**59.107**	**60.201**	60.569	**60.384**
DEL	Sniffles	55.628	64.308	59.654	54.320	64.977	59.173	57.637	65.192	61.182
	PBSV	**63.873**	62.507	63.182	**63.161**	63.064	63.112	65.006	61.983	63.459
	SVIM	53.081	65.714	58.726	51.097	66.439	57.766	55.699	66.379	60.572
	cuteSV	54.122	**67.872**	60.222	51.353	**69.149**	58.937	55.280	**68.011**	60.988
	cnnLSV	63.460	64.843	**64.144**	62.813	65.416	**64.088**	**65.299**	64.797	**65.047**
INV	Sniffles	9.091	7.009	7.916	6.278	7.273	6.739	9.420	6.667	7.808
	PBSV	32.692	9.346	14.536	26.923	7.273	11.452	**32.653**	9.333	14.517
	SVIM	10.000	**10.280**	10.138	6.020	**9.545**	7.384	9.548	**10.667**	10.076
	cuteSV	27.907	6.542	10.599	25.000	6.364	10.145	30.952	6.667	10.970
	cnnLSV	**36.170**	9.813	**15.438**	**27.451**	7.273	**11.499**	31.481	9.778	**14.921**
All	Sniffles	47.653	62.141	53.941	40.856	62.839	49.517	47.844	62.647	54.254
	PBSV	55.814	57.454	56.623	55.239	57.768	56.475	56.924	56.746	56.835
	SVIM	52.143	**66.584**	58.486	45.421	63.713	53.034	49.536	63.356	55.600
	cuteSV	47.653	62.931	54.236	51.476	**68.477**	58.772	53.306	**65.419**	58.745
	cnnLSV	**59.889**	61.781	**60.821**	**60.562**	61.613	**61.083**	**62.371**	62.329	**62.350**

The values in bold represent the best results

We can see from Table 3 that for detecting INS, DUP, DEL, and INV, our method cnnLSV achieved higher F1-scores than the other four methods on all three datasets overall. This means that cnnLSV has a better comprehensive detection ability for various types of SV. And the existing detection methods are often good at detecting specific types of structural variants. For example, cuteSV had a good performance in detecting INS/DUP, but PBSV is good at detecting DEL and INV. In addition, we can also find that cnnLSV can better balance precision and recall to obtain a higher F1-scores, especially when detecting INS/DUP and DEL. Due to the lack of TRA in the ground truth set, we did not evaluate the detection results of TRA. The detailed detection results on HG00514, HG00733, and NA19240 datasets for all five methods are provided in the supplementary materials (See: Table S6, S7, and S8 in Additional file 1). The effectiveness of cnnLSV in each stage can also be seen in the supplementary materials (See: Table S9 in Additional file 1).

#### Case study

The dataset HG002 is real sequence alignment data from an Ashkenazim son. The cnnLSV detected SVs in HG002 dataset and outputted detailed information for each variant. Table 4 lists some key information about several SVs, where “CHROM”, “POS”, “ID”, “ALT”, “REF”, “QUAL”, and “INFO” represent the chromosome, position, identity, base, sequence, confidence score, and detailed information of each variant respectively, and “FILTER” indicates whether the variant is filtered out.

**Table 4:** Case study of cnnLSV on the HG002 dataset

Chrom	POS	ID	ALT	Ref	Qual	Filter	Info
1	934137	INS.177	C	CGCGGAGCGGAGGGC	60	PASS	SVTYPE=INS;
				GCGGAGCGGAGGGGA			END=934137;
				GGGCGCGGAGCGGAG			SVLEN=59;
				GGGGAGGGCGCCGGA			SUPPORT=5;
18	7819365	INS.590523	A	AGAACGATAGAAG	60	PASS	SVTYPE=INS;
				CGCTGGATGTTGA			END=7819365;
				GGGAGGGTGGAGC			SVLEN=53;
				ACACTGGCAGAAG			SUPPORT=2;
5	56131176	DEL.3813	G	GTTGACAGGAAGGCAG	60	PASS	SVTYPE=DEL;
				GGAAGAGGAGACAGGA			END=56131239;
				AGGCAGGGAAGAGGAG			SVLEN=64;
				ACAAGGAGGCAGGGAA			SUPPORT=8;
9	5441971	DEL.4918	A	ATACGTGTGTATATAC	60	PASS	SVTYPE=DEL;
				ACACGTATATACATGT			END=5442033;
				GTGTATATACATGTGT			SVLEN=63;
				GTATATACACACGTA			SUPPORT=3;

We can see from Table 4 that cnnLSV can output the chromosome, start and end points, type, sequence, and length of each SV. In addition, cnnLSV also provides the confidence score and the number of supporting reads of each SV for subsequent genomic analysis.

## Discussion

According to the characteristics of the long reads and multiple types of SV, we design an encoding strategy for four kinds of structural variants and propose a deep learning-based multi-type SV detection method using long-read alignment information. In the image encoding stage, our method cnnLSV can automatically adjust the images from different variants to a uniform size according to the length of each variant and the coverage of the dataset for training the filtering model. And in the training model phase, cnnLSV converts the images in training set into one-dimensional arrays, and executes the principal component analysis and *k*-means clustering to eliminate the incorrectly labeled images to improve the filtering performance of the model. The experimental results on simulated and real datasets show that the overall performance of our method is better than that of other existing methods on detecting INS, DEL, INV, and DUP.

At present, cnnLSV just combines the TRA from detection results of existing detection methods, and does not support filtering for TRA temporarily because the read stacking method is not suitable for TRA. Since cnnLSV encodes all variant fragments in long reads, it will take more time and memory, especially when detecting large SVs on high coverage datasets. In the future, we will further investigate the detection of TRA, optimization of specific implementation of the encoding method to reduce memory usage, and parallel processing of cnnLSV to accelerate the detection.

## Conclusion

In this paper, our proposed method cnnLSV detects SV by encoding long-read alignment information and CNN, and uses PCA and *k*-means algorithms to eliminate mislabeled samples to improve the performance of model in the training model phase. The experimental results on simulated and real datasets show that the detection performance of cnnLSV is overall better than existing detection algorithms. We will continue to investigate the detection of TRAs and the optimize the performance of cnnLSV in terms of detection speed and memory usage in the future.

## Supplementary Information

**Additional file 1.** Supplementary information.

## Data Availability

The datasets used in this paper are all publicly available. The nine PacBio CLR datasets HG00512, HG00513, HG00514, HG00731, HG00732, HG00733, NA19238, NA19239, and NA19240 are downloaded from http://ftp.1000genomes.ebi.ac.uk/vol1/ftp/data_collections/hgsv_sv_discovery/working/20160905_smithm_pacbio_aligns/, and their ground truth sets are downloaded from https://ftp.ncbi.nlm.nih.gov/pub/dbVar/data/Homo_sapiens/by_study/vcf/nstd152.GRCh37.variant_call.vcf.gz. The two datasets HG002 CLR and HG002 CCS and corresponding ground truth sets are downloaded from https://ftp.ncbi.nih.gov/giab/ftp/data/AshkenazimTrio/. The program of cnnLSV can be downloaded from https://github.com/mhuidong/cnnLSV.

## Abbreviations

bp: Base pair
SNP: Single nucleotide polymorphism
Indel: Small insertion and deletion
SV: Structural variant
NGS: Next-generation sequencing
TGS: Third-generation sequencing
PCA: Principal component analysis
CNN: Convolutional neural network
VCF: Variant call format
