# Supplementary material for: cnnLSV: detecting structural variants by encoding long-read alignment information and convolutional neural network
Source: BMC Bioinformatics. 2023 Mar 28;24:119. doi: 10.1186/s12859-023-05243-x (PMC10045035; doi:10.1186/s12859-023-05243-x)
Supplement: Supplementary file 1 — Additional file 1. Supplementary information. [file 12859_2023_5243_MOESM1_ESM.pdf]

Supplementary information

The detailed detection results on the simulated datasets

The detailed detection results on the simulated datasets with 30×, 20×, and 10× coverage are shown in Table S1, S2 and S3, respectively. The number of correct detections and the number of incorrect detections in the results of the detection methods are recorded as *TP-call* and *FP*, respectively. And the number of detected SVs and the number of undetected SVs in the ground truth set are denoted as *TP-base* and *FN*, respectively.

Table S1 Detection results on 30x simulated datasets

Type	Method	*TP-call*	*TP-base*	*FP*	*FN*	*Pre* (%)	*Rec* (%)	*F*1 (%)
INS	Sniffles	1606	1643	213	392	88.290	80.737	84.345
	PBSV	1559	1602	502	433	75.643	78.722	77.152
	SVIM	1973	1845	849	190	69.915	90.663	78.949
	cuteSV	1629	1641	314	394	83.839	80.639	82.208
	cnnLSV	3142	1682	442	353	87.667	82.654	85.087
DEL	Sniffles	1085	1092	61	102	94.677	91.457	93.039
	PBSV	1043	1064	39	130	96.396	89.112	92.611
	SVIM	1110	1121	66	73	94.388	93.886	94.136
	cuteSV	1075	1096	53	98	95.301	91.792	93.514
	cnnLSV	1093	1109	45	85	96.046	92.881	94.437
INV	Sniffles	3	3	2	3	60.000	50.000	54.545
	PBSV	3	3	1	3	75.000	50.000	60.000
	SVIM	3	3	2	3	60.000	50.000	54.545
	cuteSV	7	3	4	3	63.636	50.000	56.000
	cnnLSV	3	3	1	3	75.000	50.000	60.000
DUP	Sniffles	693	652	221	229	75.821	74.007	74.903
	PBSV	533	516	263	365	66.960	58.570	62.484
	SVIM	665	623	235	258	73.889	70.715	72.267
	cuteSV	784	700	272	181	74.242	79.455	76.760
	cnnLSV	909	703	233	178	79.597	79.796	79.696
TRA	Sniffles	1156	679	316	329	78.533	67.361	72.519
	PBSV	2625	889	117	119	95.733	88.194	91.809
	SVIM	2561	868	117	140	95.631	86.111	90.622
	cuteSV	1484	938	80	70	94.885	93.056	93.961
	cnnLSV	1686	891	78	117	95.578	88.393	91.845

Table S2 Detection results on 20x simulated datasets

Type	Method	*TP-call*	*TP-base*	*FP*	*FN*	*Pre* (%)	*Rec* (%)	*F1* (%)
INS	Sniffles	1601	1638	212	397	88.307	80.491	84.218
	PBSV	1591	1610	468	425	77.271	79.115	78.182
	SVIM	1929	1813	876	222	68.770	89.091	77.623
	cuteSV	1671	1675	322	360	83.843	82.310	83.069
	cnnLSV	2142	1709	385	326	84.765	83.980	84.371
DEL	Sniffles	1101	1102	70	92	94.022	92.295	93.150
	PBSV	1049	1064	33	130	96.950	89.112	92.866
	SVIM	1114	1124	71	70	94.008	94.137	94.073
	cuteSV	1088	1106	63	88	94.526	92.630	93.569
	cnnLSV	1175	1118	65	76	94.758	93.635	94.193
INV	Sniffles	3	3	3	3	50.000	50.000	50.000
	PBSV	3	3	1	3	75.000	50.000	60.000
	SVIM	3	3	2	3	60.000	50.000	54.545
	cuteSV	6	3	4	3	60.000	50.000	54.545
	cnnLSV	3	3	1	3	75.000	50.000	60.000
DUP	Sniffles	691	644	211	237	76.608	73.099	74.812
	PBSV	524	512	231	369	69.404	58.116	63.260
	SVIM	668	620	268	261	71.368	70.375	70.868
	cuteSV	744	684	278	197	72.798	77.639	75.141
	cnnLSV	913	694	299	187	75.330	78.774	77.014
TRA	Sniffles	1088	671	384	337	73.913	66.567	70.048
	PBSV	2589	876	115	132	95.747	86.905	91.112
	SVIM	2586	872	134	136	95.074	86.508	90.589
	cuteSV	1482	938	68	70	95.613	93.056	94.317
	cnnLSV	1721	884	80	124	95.558	87.698	91.460

Table S3 Detection results on 10x simulated datasets

Type	Method	*TP-call*	*TP-base*	*FP*	*FN*	*Pre* (%)	*Rec* (%)	*F1* (%)
INS	Sniffles	1602	1624	257	411	86.175	79.803	82.867
	PBSV	1420	1442	353	593	80.090	70.860	75.193
	SVIM	1806	1718	863	317	67.666	84.423	75.121
	cuteSV	1621	1606	374	429	81.253	78.919	80.069
	cnnLSV	1766	1667	419	368	80.824	81.916	81.366
DEL	Sniffles	1071	1061	66	133	94.195	88.861	91.450
	PBSV	975	989	35	205	96.535	82.831	89.159
	SVIM	1076	1086	79	108	93.160	90.955	92.044
	cuteSV	1056	1067	72	127	93.617	89.363	91.441
	cnnLSV	1154	1085	76	109	93.821	90.871	92.323
INV	Sniffles	3	3	2	3	60.000	50.000	54.545
	PBSV	3	3	1	3	75.000	50.000	60.000
	SVIM	3	3	1	3	75.000	50.000	60.000
	cuteSV	6	3	4	3	60.000	50.000	54.545
	cnnLSV	3	3	1	3	75.000	50.000	60.000
DUP	Sniffles	638	602	228	279	73.672	68.331	70.901
	PBSV	492	483	205	398	70.588	54.824	61.715
	SVIM	611	573	319	308	65.699	65.040	65.368
	cuteSV	691	636	310	245	69.031	72.191	70.575
	cnnLSV	834	649	321	232	72.208	73.666	72.930
TRA	Sniffles	874	592	587	416	59.822	58.730	59.271
	PBSV	2277	819	109	189	95.432	81.250	87.772
	SVIM	2458	845	122	163	95.271	83.829	89.185
	cuteSV	1463	924	73	84	95.247	91.667	93.423
	cnnLSV	1754	858	74	150	95.952	85.119	90.211

The detailed detection results on the HG002 datasets

The detailed detection results on the PacBio datasets HG002 CLR and HG002 CCS are shown in Table S4.

Table S4 Detection results on HG002 CLR and HG002 CCS datasets

Coverage	Method	*TP-call*	*TP-base*	*FP*	*FN*	*Pre* (%)	*Rec* (%)	*F1* (%)
				HG002 CLR				
69×	Sniffles	8606	8606	514	1035	94.364	89.265	91.744
	PBSV	8483	8443	463	1198	94.825	87.574	91.055
	SVIM	8970	8860	474	781	94.981	91.899	93.415
	cuteSV	8831	8826	442	815	95.233	91.547	93.354
	cnnLSV	9039	9005	379	636	95.976	93.403	94.672
40×	Sniffles	8699	8694	642	947	93.127	90.177	91.628
	PBSV	8374	8332	461	1309	94.782	86.423	90.410
	SVIM	8620	8518	421	1123	95.343	88.352	91.715
	cuteSV	8788	8770	438	871	95.253	90.966	93.060
	cnnLSV	8875	8846	417	795	95.512	91.754	93.595
30×	Sniffles	8562	8550	774	1091	91.710	88.684	90.171
	PBSV	8134	8100	428	1541	95.001	84.016	89.172
	SVIM	7991	7926	309	1715	96.277	82.211	88.690
	cuteSV	8558	8535	456	1106	94.941	88.528	91.623
	cnnLSV	8658	8620	442	1021	95.143	89.410	92.187
20×	Sniffles	7461	7454	575	2187	92.845	77.316	84.372
	PBSV	7450	7416	357	2225	95.427	76.921	85.181
	SVIM	8955	8713	1515	928	85.530	90.374	87.886
	cuteSV	7480	7469	296	2172	96.193	77.471	85.823
	cnnLSV	8564	8508	661	1133	92.835	88.248	90.483
10×	Sniffles	5562	5556	596	4085	90.322	57.629	70.363
	PBSV	4794	4781	154	4860	96.888	49.590	65.603
	SVIM	7178	7104	638	2537	91.837	73.685	81.766
	cuteSV	5541	5532	209	4109	96.365	57.380	71.930
	cnnLSV	7125	7101	606	2540	92.161	73.654	81.875
				HG002 CCS				
28×	Sniffles	9072	9041	573	600	94.059	93.777	93.918
	PBSV	8132	8115	554	1526	93.622	84.172	88.646
	SVIM	8957	8944	642	697	93.312	92.770	93.040
	cuteSV	9013	9004	478	637	94.964	93.393	94.172
	cnnLSV	9057	9031	491	610	94.858	93.673	94.261
10×	Sniffles	8553	8520	537	1121	94.092	88.373	91.143
	PBSV	7289	7282	346	2359	95.468	75.532	84.338
	SVIM	8726	8710	739	931	92.192	90.343	91.258
	cuteSV	8401	8389	448	1252	94.937	87.014	90.803
	cnnLSV	8787	8761	586	880	93.748	90.872	92.288

The detailed detection results of methods for different lengths of SVs

The detailed detection results of methods on 69× HG002 CLR dataset for different lengths of SVs

Table S5 Detection results of tools in detecting different lengths of SVs

SV length	Method	*TP-call*	*TP-base*	*FP*	*FN*	*Pre* (%)	*Rec* (%)	*F1* (%)
$[50, 100)$	Sniffles	2744	2765	314	190	89.732	93.570	91.611
	PBSV	2442	2489	232	466	91.324	84.230	87.634
	SVIM	2854	2817	240	138	92.243	95.330	93.761
	cuteSV	2770	2775	235	180	92.180	93.909	93.036
	cnnLSV	2712	2703	204	252	93.004	91.472	92.232
$[100, 200)$	Sniffles	1600	1590	89	43	94.731	97.367	96.031
	PBSV	1397	1399	90	234	93.948	85.671	89.618
	SVIM	1618	1597	110	36	93.634	97.795	95.670
	cuteSV	1603	1600	85	33	94.964	97.979	96.448
	cnnLSV	1603	1587	64	46	96.161	97.183	96.669
$[200, 500)$	Sniffles	2983	2992	77	94	97.484	96.954	97.218
	PBSV	2931	2924	62	162	97.928	94.750	96.313
	SVIM	3061	3033	92	53	97.082	98.283	97.679
	cuteSV	3014	3010	83	76	97.320	97.537	97.429
	cnnLSV	3036	3031	67	55	97.841	98.218	98.029
$[500, 1000)$	Sniffles	590	574	20	113	96.721	83.552	89.655
	PBSV	618	608	37	79	94.351	88.501	91.332
	SVIM	635	619	25	68	96.212	90.102	93.057
	cuteSV	577	581	31	106	94.901	84.571	89.439
	cnnLSV	621	625	32	62	95.100	90.975	92.992
$[1000, 2000)$	Sniffles	272	276	2	256	99.270	51.880	68.146
	PBSV	407	407	26	125	93.995	76.504	84.352
	SVIM	381	377	5	155	98.705	70.865	82.499
	cuteSV	325	330	6	202	98.187	62.030	76.029
	cnnLSV	422	428	7	104	98.368	80.451	88.512
$[2000, 5000)$	Sniffles	299	291	10	196	96.764	59.754	73.883
	PBSV	442	438	12	49	97.357	89.938	93.501
	SVIM	305	306	2	181	99.349	62.834	76.980
	cuteSV	382	372	2	115	99.479	76.386	86.416
	cnnLSV	443	431	3	56	99.327	88.501	93.602
$[5000, 10000)$	Sniffles	95	95	2	113	97.938	45.673	62.295
	PBSV	154	154	2	54	98.718	74.038	84.615
	SVIM	89	89	0	119	100.000	42.788	59.933
	cuteSV	132	130	0	78	100.000	62.500	76.923
	cnnLSV	172	170	2	38	98.851	81.731	89.479
$[10000, +\infty)$	Sniffles	23	23	0	30	100.000	43.396	60.526
	PBSV	24	24	2	29	92.308	45.283	60.759
	SVIM	22	22	0	31	100.000	41.509	58.667
	cuteSV	28	28	0	25	100.000	52.830	69.136
	cnnLSV	30	30	0	23	100.000	56.604	72.289

The detailed detection results on HG00514, HG00733, and NA19240 datasets

The detailed detection results on the real datasets HG00514, HG00733, and NA19240 are shown in Table S6, S7 and S8, respectively.

Table S6 Detection results on HG00514 dataset

Type	Method	*TP-call*	*TP-base*	*FP*	*FN*	*Pre* (%)	*Rec* (%)	*F1* (%)
INS/DUP	Sniffles	8221	10619	10775	6808	43.278	60.934	50.610
	PBSV	7142	9350	6976	8077	50.588	53.652	52.075
	SVIM	8341	10658	10378	6769	44.559	61.158	51.555
	cuteSV	10279	11537	9884	5890	50.980	66.202	57.602
	cnnLSV	8315	10414	6136	7013	57.539	59.758	58.628
DEL	Sniffles	6153	9744	4908	5408	55.628	64.308	59.654
	PBSV	5944	9471	3362	5681	63.873	62.507	63.182
	SVIM	6288	9957	5558	5195	53.081	65.714	58.726
	cuteSV	6703	10284	5682	4868	54.122	67.872	60.222
	cnnLSV	6233	9825	3589	5327	63.460	64.843	64.144
INV	Sniffles	12	15	120	199	9.091	7.009	7.916
	PBSV	17	20	35	194	32.692	9.346	14.536
	SVIM	17	22	153	192	10.000	10.280	10.138
	cuteSV	12	14	31	200	27.907	6.542	10.599
	cnnLSV	17	21	30	193	36.170	9.813	15.438
All	Sniffles	14386	20378	15803	12415	47.653	62.141	53.941
	PBSV	13103	18841	10373	13952	55.814	57.454	56.623
	SVIM	16994	21835	15597	10958	52.143	66.584	58.486
	cuteSV	14646	20637	16089	12156	47.653	62.931	54.236
	cnnLSV	14565	20260	9755	12533	59.889	61.781	60.821

Table S7 Detection results on HG00733 dataset

Type	Method	*TP-call*	*TP-base*	*FP*	*FN*	*Pre* (%)	*Rec* (%)	*F1* (%)
INS/DUP	Sniffles	8582	11122	16003	6895	34.907	61.731	44.596
	PBSV	7408	9722	7333	8295	50.254	53.960	52.041
	SVIM	8803	11188	11874	6829	42.574	62.097	50.515
	cuteSV	11202	12373	10503	5644	51.610	68.674	58.932
	cnnLSV	8516	10648	5890	7369	59.114	59.100	59.107
DEL	Sniffles	6211	9781	5223	5272	54.320	64.977	59.173
	PBSV	5975	9493	3485	5560	63.161	63.064	63.112
	SVIM	6360	10001	6087	5052	51.097	66.439	57.766
	cuteSV	6830	10409	6470	4644	51.353	69.149	58.937
	cnnLSV	6292	9847	3725	5206	62.813	65.416	64.088
INV	Sniffles	14	16	209	204	6.278	7.273	6.739
	PBSV	14	16	38	204	26.923	7.273	11.452
	SVIM	18	21	281	199	6.020	9.545	7.384
	cuteSV	12	14	36	206	25.000	6.364	10.145
	cnnLSV	14	16	37	204	27.451	7.273	11.499
All	Sniffles	14807	20919	21435	12371	49.391	54.482	51.811
	PBSV	13397	19231	10856	14059	63.918	48.794	55.342
	SVIM	15181	21210	18242	12080	53.762	55.688	54.708
	cuteSV	18044	22796	17009	10494	57.269	63.228	60.101
	cnnLSV	14822	20511	9652	12779	68.001	53.701	60.011

Table S8 Detection results on NA19240 dataset

Type	Method	*TP-call*	*TP-base*	*FP*	*FN*	*Pre* (%)	*Rec* (%)	*F1* (%)
INS/DUP	Sniffles	9058	11874	12397	7608	42.219	60.949	49.883
	PBSV	7660	10222	7315	9260	51.152	52.469	51.802
	SVIM	9183	11919	10884	7563	45.762	61.180	52.359
	cuteSV	10590	12412	9790	7070	51.963	63.710	57.240
	cnnLSV	8758	11172	5790	7273	60.201	60.569	60.384
DEL	Sniffles	7415	11702	5450	6248	57.637	65.192	61.182
	PBSV	7048	11126	3794	6824	65.006	61.983	63.459
	SVIM	7565	11915	6017	6035	55.699	66.379	60.572
	cuteSV	7931	12208	6416	5742	55.280	68.011	60.988
	cnnLSV	7416	11631	3941	6319	65.299	64.797	65.047
INV	Sniffles	13	15	125	210	9.420	6.667	7.808
	PBSV	16	21	33	204	32.653	9.333	14.517
	SVIM	19	24	180	201	9.548	10.667	10.076
	cuteSV	13	15	29	210	30.952	6.667	10.970
	cnnLSV	17	22	37	203	31.481	9.778	14.921
All	Sniffles	16486	23591	17972	14066	56.760	53.960	55.325
	PBSV	14724	21369	11142	16288	65.729	47.478	55.132
	SVIM	16767	23858	17081	13799	58.277	54.855	56.514
	cuteSV	18534	24635	16235	13022	60.276	58.734	59.495
	cnnLSV	16191	22825	9768	13795	70.030	53.995	60.976

The effectiveness of cnnLSV

Since cnnLSV is based on the callsets of existing methods to get higher quality detection results, we only discuss the runtime of cnnLSV itself here. The runtime of each phase of cnnLSV is shown in Table S9.

Table S9 Runtime of training model and detecting SVs in cnnLSV

Training model				
Dataset	Runtime (Seconds)			
	Generating initial *Img*	Eliminating incorrectly labeled images	Balancing training sets	Training the filtering model
HG00512	3409.58	6998.56	1150.87	
HG00513	2997.06	3238.48	1818.32	
HG00731	6457.07	5850.85	806.08	6833.63
HG00732	6670.10	6548.06	979.13	(25 epochs)
NA19238	4332.74	4824.27	1128.10	
NA19239	4402.04	4683.84	932.85	

Detecting SVs		
Dataset	Number of filtered SVs	Runtime (Seconds)
HG002 CLR 69×	102670	29722.35
HG002 CLR 40×	98781	20087.15
HG002 CLR 30×	95182	19816.28
HG002 CLR 20×	96587	16101.24
HG002 CLR 10×	66286	10382.69
HG002 CCS 28×	107980	13586.09
HG002 CCS 10×	98430	13068.25
HG00514	115535	52254.94
HG00733	127252	69660.06
NA19240	127691	56366.49

From Table S9, we can see that the training model phase spent long runtime. This is because we use sufficiently large data samples from six real datasets to train the model. Our trained model can support the detection of PacBio datasets, which is directly available at https://github.com/mhuidong/cnnLSV. In the detecting vari-

ants stage, since cnnLSV filtered the callset merged from the results of four existing methods, this definitely increases the overall detection time. Besides, the runtime is also closely related to the coverages of datasets. In the future, one of our works is to further investigate the acceleration of cnnLSV.
